# Rapid Sequestration of *Leishmania mexicana* by Neutrophils Contributes to the Development of Chronic Lesion

**DOI:** 10.1371/journal.ppat.1004929

**Published:** 2015-05-28

**Authors:** Benjamin P. Hurrell, Steffen Schuster, Eva Grün, Manuel Coutaz, Roderick A. Williams, Werner Held, Bernard Malissen, Marie Malissen, Shida Yousefi, Hans-Uwe Simon, Andreas J. Müller, Fabienne Tacchini-Cottier

**Affiliations:** 1 Department of Biochemistry, WHO-Immunology Research and Training Center, University of Lausanne, Epalinges, Switzerland; 2 School of Science and Sport, University of the West of Scotland, Paisley, United Kingdom; 3 Ludwig Center for Cancer Research, University of Lausanne, Epalinges, Switzerland; 4 Centre d'Immunologie de Marseille-Luminy (CIML) Aix Marseille Université, UM2, Marseille, France; 5 INSERM U1104, Marseille, France; 6 CNRS UMR7280, Marseille, France; 7 Institute of Pharmacology, University of Bern, Bern, Switzerland; 8 Otto-von-Guericke-University Magdeburg and Helmholtz Centre for Infection Research- Braunschweig, Magdeburg, Germany; Imperial College London, UNITED KINGDOM

## Abstract

The protozoan *Leishmania mexicana* parasite causes chronic non-healing cutaneous lesions in humans and mice with poor parasite control. The mechanisms preventing the development of a protective immune response against this parasite are unclear. Here we provide data demonstrating that parasite sequestration by neutrophils is responsible for disease progression in mice. Within hours of infection *L*. *mexicana* induced the local recruitment of neutrophils, which ingested parasites and formed extracellular traps without markedly impairing parasite survival. We further showed that the *L*. *mexicana*-induced recruitment of neutrophils impaired the early recruitment of dendritic cells at the site of infection as observed by intravital 2-photon microscopy and flow cytometry analysis. Indeed, infection of neutropenic *Genista* mice and of mice depleted of neutrophils at the onset of infection demonstrated a prominent role for neutrophils in this process. Furthermore, an increase in monocyte-derived dendritic cells was also observed in draining lymph nodes of neutropenic mice, correlating with subsequent increased frequency of IFNγ-secreting T helper cells, and better parasite control leading ultimately to complete healing of the lesion. Altogether, these findings show that *L*. *mexicana* exploits neutrophils to block the induction of a protective immune response and impairs the control of lesion development. Our data thus demonstrate an unanticipated negative role for these innate immune cells in host defense, suggesting that in certain forms of cutaneous leishmaniasis, regulating neutrophil recruitment could be a strategy to promote lesion healing.

## Author Summary

Infection with the protozoan *Leishmania* parasites causes a spectrum of diseases ranging from cutaneous to visceral forms that are fatal if left untreated. Among the different *Leishmania* species, *Leishmania mexicana* causes chronic cutaneous lesions in humans. To study this disease, we used a murine model. Following infection with *Leishmania mexicana*, most mouse species including C57BL/6 develop chronic non-healing lesion. Within hours of infection, neutrophils are recruited locally and they ingest the parasites. Although neutrophils are leukocytes that are able to rapidly kill pathogens using an arsenal of molecules, several microorganisms including some, but not all, *Leishmania* species are able to survive within these cells. Here, we show that *L*. *mexicana* elicits the rapid recruitment of neutrophils at the site of infection, survives within these cells and uses them to its advantage. Furthermore, transient parasite sequestration by neutrophils delays recruitment of other leukocytes such as monocytes, contributing to the impaired development of a protective immune response against the parasite and chronic lesion development. Thus, we describe a previously unanticipated pathogenic role for neutrophils in chronic lesion development. More importantly, our data suggest that in certain forms of cutaneous leishmaniasis, regulating neutrophil recruitment could be a strategy to promote lesion healing.

## Introduction

*Leishmania* species (spp) are intracellular protozoan parasites that cause leishmaniases, a spectrum of diseases ranging from cutaneous lesions to deadly visceral forms. Following infection, the promastigote form of the parasite is inoculated in mammalian hosts by sand flies. The parasites then transform into the amastigote form and multiply within macrophages, the parasite’s final host cells. Protection against the disease has been linked to secretion of IL-12 by innate cells, promoting the differentiation of IFNγ-producing CD4^+^ Th1 cells. IFNγ induces parasite killing by macrophages correlating with resolution of infection, reviewed in [1,2]. *Leishmania (L*.*) mexicana* is a member of the New-World *Leishmania* species that cause chronic cutaneous infection in humans and mice. The events involved in the initiation of an immune response to *L*. *mexicana* infection are still poorly understood and are studied in experimental models, reviewed in [3]. Following *L*. *mexicana* infection, most mouse strains develop a progressive non-healing chronic lesion with parasite persistence. Susceptibility of C57BL/6 mice to *L*. *mexicana* infection correlates *in vivo* with poor recruitment of monocytes and DCs, as well as low expansion of parasite responding Th1 cells [4,5]. Failure to control lesion size and parasite load in these mice has been linked to low IL-12 release by innate immune cells such as macrophages and dendritic cells (DCs) correlating with poor development of Th1 cells [6–10].

Neutrophils are part of the first line of defense against pathogens. They rapidly phagocytose microorganisms and kill them by different mechanisms including the production of radical oxygen species and antibacterial peptides. In addition, neutrophils can form extracellular traps (NETs) that are DNA structures coated with antimicrobial molecules [11]. NETs sequester microorganisms, immobilize them and in some cases kill them, reviewed in [12,13].

Neutrophils are rapidly and massively recruited to the site of infection in experimental models of cutaneous (*L*. *major*, *L*. *amazonensis*) or visceral (*L*. *infantum*) leishmaniasis [14–19]. Interestingly, *L*. *major* parasites can escape killing within neutrophils prior to their uptake by macrophages either by phagocytosis of apoptotic parasitized neutrophils and/or of live parasites released by neutrophils [16,20]. In addition, some *Leishmania* spp induce NET formation *in vitro* that traps them, and may or may not reduce parasite survival [21,22]. Different *Leishmania*-spp-specific features may explain the inconsistent effects of neutrophils on *Leishmania spp* survival.

Here, we investigated the role of neutrophils following *L*. *mexicana* infection, both at the onset of infection using transient neutrophil depletion and during acute and chronic phases of the disease using the neutropenic *Genista* mice. Our results show that early sequestration of parasites by neutrophils has a major negative impact on disease progression.

## Results

### Neutropenia leads to control of lesion development following *L*. *mexicana* infection

**Fig 1:**
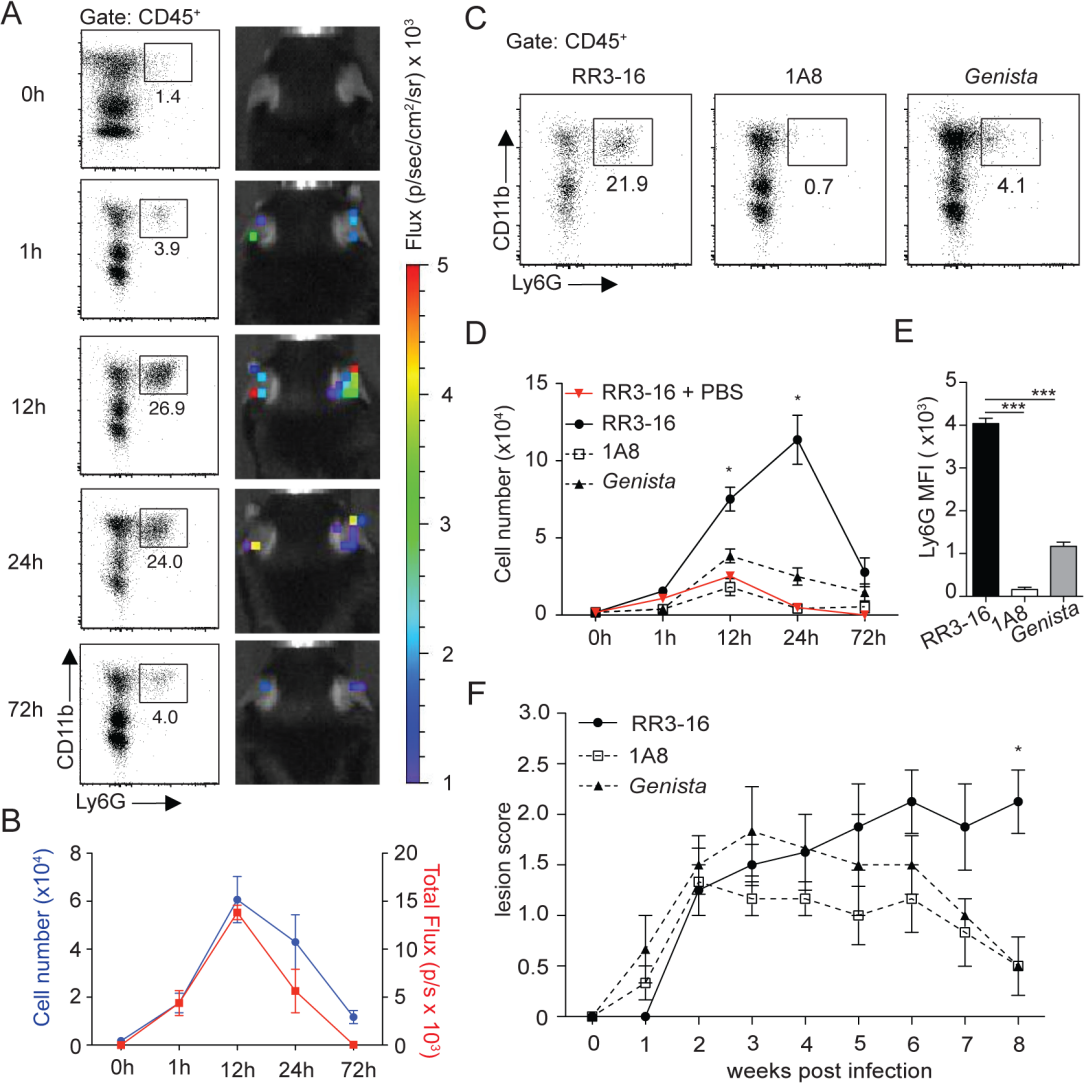
Kinetics of neutrophil recruitment and outcome on disease development following i.d. inoculation of *L*. *Mexicana*. (A) Neutrophil recruitment in the ear dermis of C57BL/6 mice inoculated with 10^6^ metacyclic *L*. *mexicana*-DsRed. Representative flow cytometry profiles of ear-derived neutrophils and chemiluminescent ear signals (MPO activity) after i.p. delivery of luminol at the indicated times after infection. (B) Quantitative analysis of the experiment described in (A). Data presented as mean ± SEM. (C) Representative flow cytometry plots showing the frequency of CD45^+^CD11b^+^Ly6G^+^ dermal cells 24 hours after infection of RR3-16-treated, 1A8 PMN-depleted C57BL/6 and *Genista* mice. (D) Quantitative flow cytometry analysis of ear neutrophil recruitment in the indicated groups. Data presented as mean ± SEM. (E) Ly6G expression intensity on CD45^+^CD11b^+^ dermal cells isolated 24 hours following parasite inoculation. Data are presented as mean MFI ± SEM. (F) Impact of neutropenia on lesion development following i.d. inoculation of 10^6^ metacyclic *L*. *mexicana* in 1A8 PMN-depleted C57BL/6 and *Genista* mice compared to RR3-16-treated C57BL/6 control mice. Ear lesion scores were measured on a weekly basis as described in the *Materials and Methods*. Results are presented as mean lesion score ± SEM. All data are representative of > 2 independent experiments and for n = 6. * p<0.05 *** p<0.001.

In order to evaluate the role of neutrophils on disease development after *L*. *mexicana* infection, we analyzed the kinetics of neutrophil recruitment during the first hours following inoculation of infectious metacyclic parasites in the ear pinna of C57BL/6 mice. Neutrophils are rapidly and massively recruited to the site of *Leishmania* infection following the bite of infected sand flies [16]. To determine the dose of parasites that would induce neutrophil recruitment following needle injection of infective stage *L*. *mexicana* metacyclic promastigotes in the ear dermis of mice, a dose curve was performed. Of the different parasite doses, injection of 10^6^ metacyclic *L*. *mexicana* elicited 24 hours p.i. the recruitment of neutrophils in the ear dermis of mice (S1 Fig) in numbers that were within the range of those observed in the ear dermis of mice 24 hours after sand fly transmission of *L*. *major* [16]. This parasite dose was thereon used in this study. Analysis of infiltrating immune cells using flow cytometry showed transient parasite-induced neutrophil recruitment peaking between 12 and 24 hours after infection (Fig 1A and 1B). To analyze neutrophil infiltration *in vivo*, myeloperoxidase (MPO) activity in the infected ear pinna was imaged by luminol-based bioluminescence [23]. Peak MPO activity was observed at similar kinetics post infection, suggesting rapid activation of extravasated dermal neutrophils (Fig 1A and 1B and S2).

To investigate the impact of early neutrophil recruitment on the control of infection we used C57BL/6 mice depleted of neutrophils during the first three days of infection using the neutrophil-depleting 1A8 mAb, and *Genista* mice that lack mature neutrophils [24]. The absence of mature neutrophils in these mice was verified by flow cytometry during the first 3 days of infection (Fig 1C and 1D) and (S3 Fig). Only a low frequency of immature neutrophils (Ly6G intermediate) was recruited to the site of infection in neutropenic mice (Fig 1E), in line with their atypical phenotype [24].

The impact of early and/or sustained neutropenia on the course of the disease was compared to that of C57BL/6 mice. In the first 3 weeks following intradermal (i.d.) inoculation of 10^6^ infectious *L*. *mexicana* promastigotes all groups of mice developed similar lesion sizes. The lesion size of C57BL/6 mice subsequently reached a plateau with persistent inflammatory lesion. In sharp contrast, the lesion size of mice with transient or sustained neutropenia decreased in size and was resolved eight weeks post infection (Fig 1F). These results reveal that the presence of neutrophils during the first days following *L*. *mexicana* infection prevents the resolution of the parasite-induced lesion.

### Rapid parasite-induced NET formation traps but does not kill *L*. *mexicana* parasites

**Fig 2:**
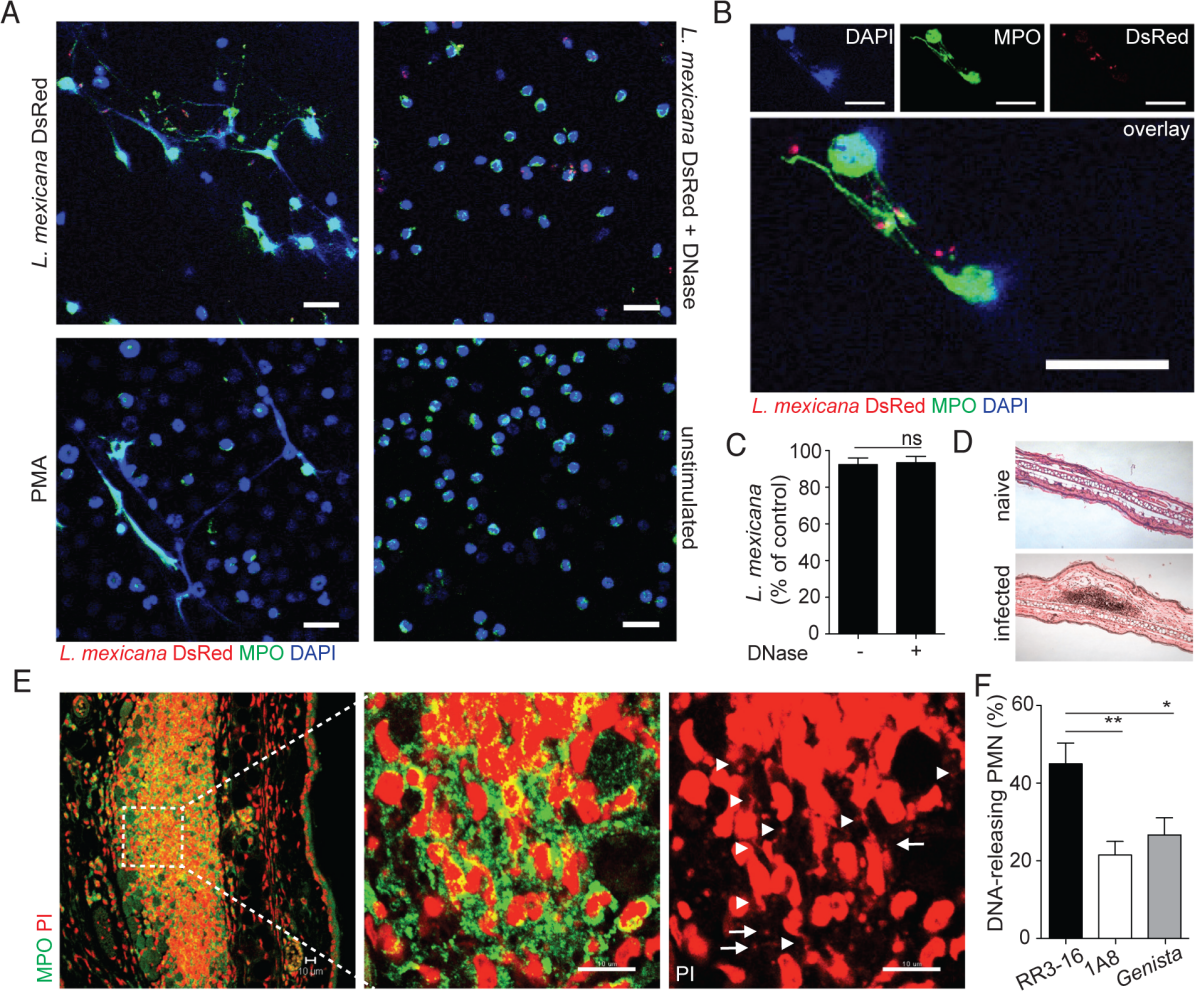
Induction of NETs by *L*. *mexicana* promastigotes. (A) Sorted mouse BM neutrophils were adhered on poly-L-lysin coverslips and incubated for 4 hours with *L*. *mexicana*-DsRed metacyclic promastigotes at a 5:1 parasite-cell MOI in absence or presence of DNase 10μg/mL. Samples were fixed, stained for MPO and DNA (DAPI) and analyzed by confocal microscopy. As controls, neutrophils were incubated with 100ng/mL PMA or medium and similarly analyzed. (B) Higher magnification of *L*. *mexicana-*DsRed-induced NETs. (C) BM neutrophils were incubated with *L*. *mexicana*-luciferase promastigotes at a 5:1 parasite-cell MOI in absence or presence of 10μg/mL DNase. After 4 hours parasite survival was determined in each group by dividing the luciferase relative light units (RLUs) obtained in presence of neutrophils from that obtained in the absence of neutrophils. (D) RR3-16-treated, 1A8 PMN-depleted C57BL/6 and *Genista* mice were inoculated i.d. with 10^6^ metacyclic *L*. *mexicana*. Ears were collected 24h later and histology performed on 4 micron sections and inflammatory cell recruitment determined by hematoxylin and eosin. (E) Ear sections were stained with polyclonal anti-MPO Ab and DNA stained with PI. Arrowheads point to neutrophil DNA fibers and arrows to parasite DNA. (F) Frequency of neutrophils producing NETs/section as determined by confocal microscopy. Data presented as mean ± SEM for n ≥ 4/group. Results representative of 3 or more independent experiments. Scale bars: 10 micron. * p<0.05 ** p<0.01, ns: non significant.

To investigate whether *L*. *mexicana* can induce NET formation, neutrophils were exposed *in vitro* to *L*. *mexicana-*DsRed and the generation of NETs was visualized by confocal microscopy for DNA and NET associated MPO. *L*. *mexicana* clearly elicited NET formation, a process that was abrogated if DNase was added (Fig 2A). NET formation was not induced in unstimulated neutrophils (Fig 2A). Of note, *L*. *mexicana*-DsRed parasites were observed either in association with NETs or detected within intact neutrophils (Fig 2A and 2B). To determine if *L*. *mexicana* parasites were killed by NETs we generated *L*. *mexicana* expressing a firefly luciferase reporter gene. Four hours after incubation with netting neutrophils the majority of *L*. *mexicana* parasites were viable luciferase-expressing *L*. *mexicana* and their frequency did not vary in co-cultures performed in presence of DNase (Fig 2C). These data demonstrate that *L*. *mexicana* are able to survive within NETs *in vitro*.

We then determined if *L*. *mexicana* triggered NET formation *in vivo*. Twenty-four hours after infection i.e. at the peak of neutrophil infiltration, immunohistology was performed on infected ears (Fig 2D). *L*. *mexicana* i.d. inoculation induced NET formation locally as judged by the presence of extracellular DNA fibers associated with MPO. In addition, parasites identified by their smaller nuclear DNA were also observed in association with NETs (Fig 2E). In neutropenic mice, the cellular infiltrate was significantly smaller as compared to C57BL/6 mice. Even though *L*. *mexicana* induced NET formation was observed in neutropenic mice at tissue depths where neutrophils were detectable, NET frequency was significantly lower as compared to C57BL/6 mice (Fig 2F). Collectively these results show that *L*. *mexicana* induces NET formation that traps parasites, but remarkably most of the trapped parasites survived.

### Absence of neutrophils improves early parasite control

**Fig 3:**
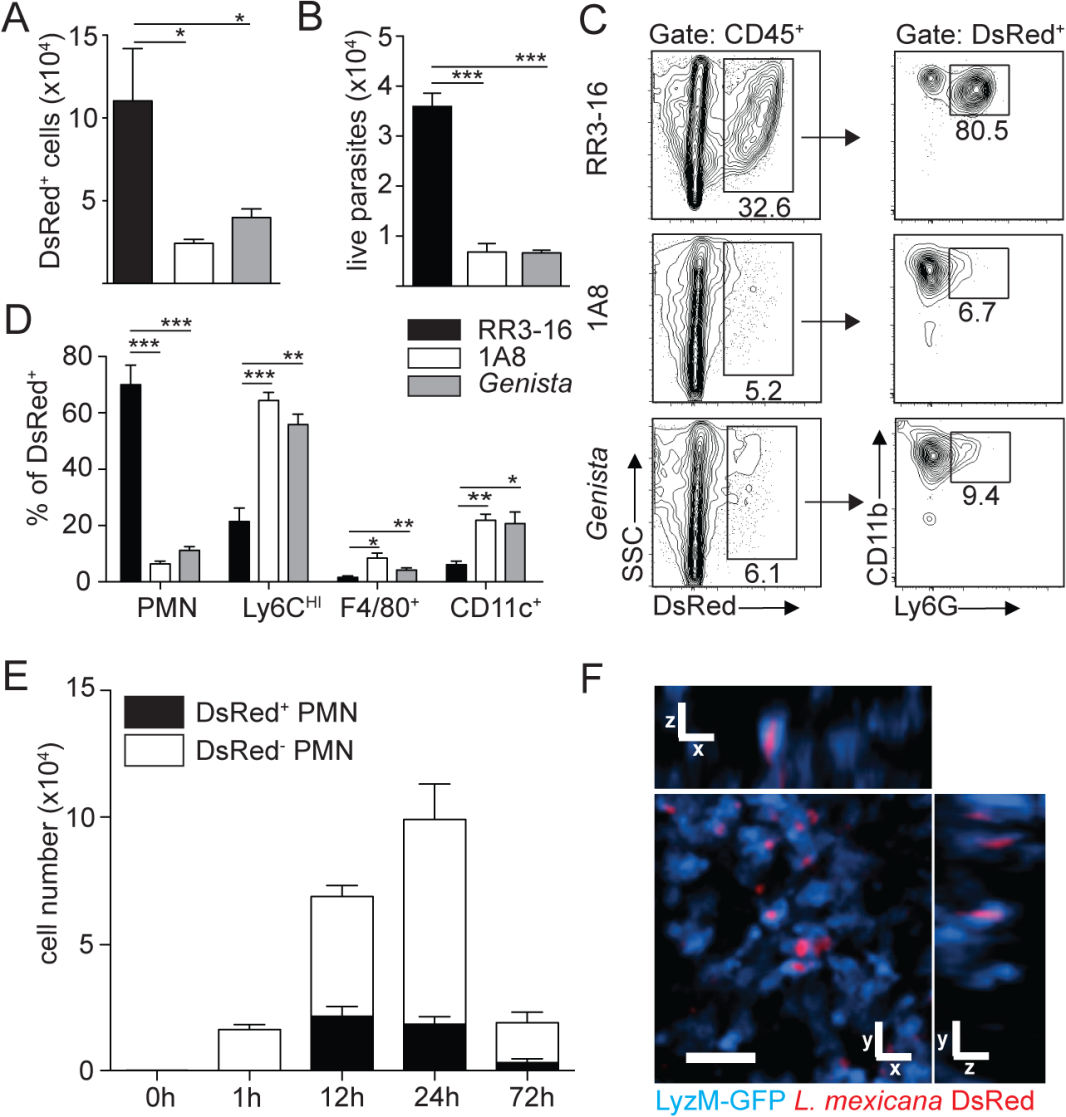
Sequestration of *L*. *mexicana* promastigotes by neutrophils impacts on early parasite control. (A) RR3-16-treated, 1A8 PMN-depleted C57BL/6 and *Genista* mice were inoculated i.d. with 10^6^ metacyclic *L*. *mexicana*-DsRed. Quantitation of ear-derived CD45^+^ DsRed^+^ cells analyzed by flow cytometry (B) and of live ear parasites analyzed by limiting dilution assay 24h p.i. Data presented as mean ± SEM (n = 6/group). (C) 24 hours following infection, ear cells in the indicated mouse groups were isolated and analyzed by flow cytometry. The frequency of CD45^+^DsRed^+^ parasitized cells and the frequency of Ly6G^*+*^CD11b^+^ neutrophils gated within this population is shown. (D) Frequency of Ly6G^+^CD11b^+^ PMN, Ly6G^-^Ly6C^high^ monocytes, Ly6G^-^CD11c^-^Ly6C^-^F4/80^+^ macrophages and Ly6G^-^CD11c^+^ dendritic cells present within CD45^+^DsRed^+^ ear cells of the indicated infected mouse groups as determined by flow cytometry. Data presented as the mean frequency of each subset ± SEM (n = 6/group). (E) C57BL/6 mice were inoculated i.d. with 10^6^ metacyclic *L*. *mexicana*-DsRed, and parasite presence in dermal neutrophil was analyzed by flow cytometry at the indicated times post infection. Data presented as the mean PMN number ± SEM (n = 6/group). (F) LyzM-GFP mice were infected i.d. with *L*. *mexicana*-DsRed and 24 hours later, a 20 micron frame projection across the x, y and z dimensions was acquired by 2-photon imaging showing the intracellular presence of parasites (red) within dermal neutrophils (bright blue). Scale bar: 20 micron. Results are representative of 2 or more independent experiments. * p<0.05 ** p<0.01 ***p<0.001, ns: non significant.

We then determined the impact of neutrophils on early parasite control at the site of infection. To this end, DsRed-expressing *L*. *mexicana* parasites were inoculated i.d. in the ear pinna. The number of live parasites in the ear pinna were significantly reduced in neutropenic mice compared to control mice as analyzed by flow cytometry (Fig 3A) and limiting dilution analysis (Fig 3B). We next determined where the parasite was located. Twenty-four hours p.i. around 80% of the infected cells found at the site of infection were CD11b^+^Ly6G^+^ neutrophils as quantified by flow cytometry (Fig 3C). Significantly lower numbers of infected cells were detected in neutropenic mice and parasites were mostly found in CD11b^+^Ly6C^high^ monocytes, followed in decreasing order by CD11b^+^CD11c^+^ dendritic cells and CD11b^+^F4/80^+^ macrophages (Fig 3D). The frequency of parasitized neutrophils peaked between 12 and 24 hours post infection (Fig 3E), and *L*. *mexicana* intracellular presence within neutrophils was confirmed *in vivo* by 2-photon intravital microscopy using LyzM-GFP mice (Fig 3F).

**Fig 4:**
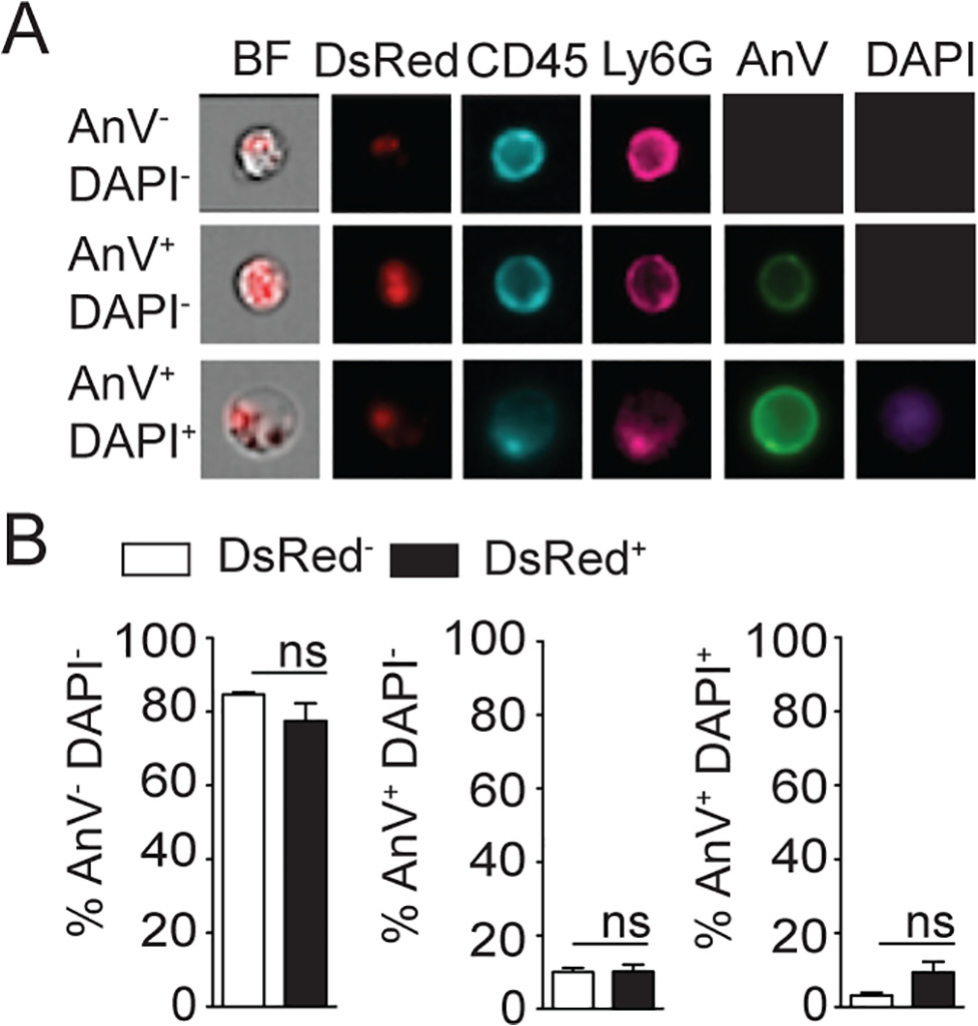
*L*. *mexicana* does not induce neutrophil apoptosis at the site of infection 24 hours after infection. (A) *L*. *mexicana*-DsRed was i.d. inoculated in C57BL/6 mice. 24 hours later ear dermal cells were isolated and apoptosis of CD45^+^Ly6G^+^CD11b^+^ neutrophil-containing or not *L*. *mexicana*-DsRed was visualized by ImageStream analysis using Annexin V and DAPI staining. Representative images are shown. (B) Frequency of AnV^-^DAPI^-^ live, AnV^+^DAPI^-^ early apoptotic or AnV^+^DAPI^+^ late apoptotic/necrotic neutrophils. Data presented as the mean frequency ± SEM (n = 4). Results are representative of 3 independent experiments. ns: non significant.

Ingestion of some microorganisms such as *Mycobacterium tuberculosis* delays neutrophil apoptosis [25], with a potential impact on the microenvironment present at the site of infection. We thus analyzed the apoptotic status of dermal neutrophils 24 hours p.i., and ImageStream imaging analysis revealed similar frequencies of AnnexinV^+^DAPI^-^ apoptotic, AnnexinV^+^DAPI^+^ apoptotic/necrotic neutrophils irrespective of the presence of *L*. *mexicana*-DsRed. This correlated with the high frequency (>80%) of live AnnexinV^-^DAPI^-^ neutrophils observed in both uninfected and infected neutrophils (Fig 4A and 4B). Altogether, these data show that presence of *L*. *mexicana* within neutrophils does not induce neutrophil apoptosis and does also not lead to parasite killing. The presence of neutrophils thus improves parasite survival.

### Neutropenia impacts cellular recruitment and changes cell dynamics at the site of *L*. *mexicana* inoculation and in draining lymph nodes

**Fig 5:**
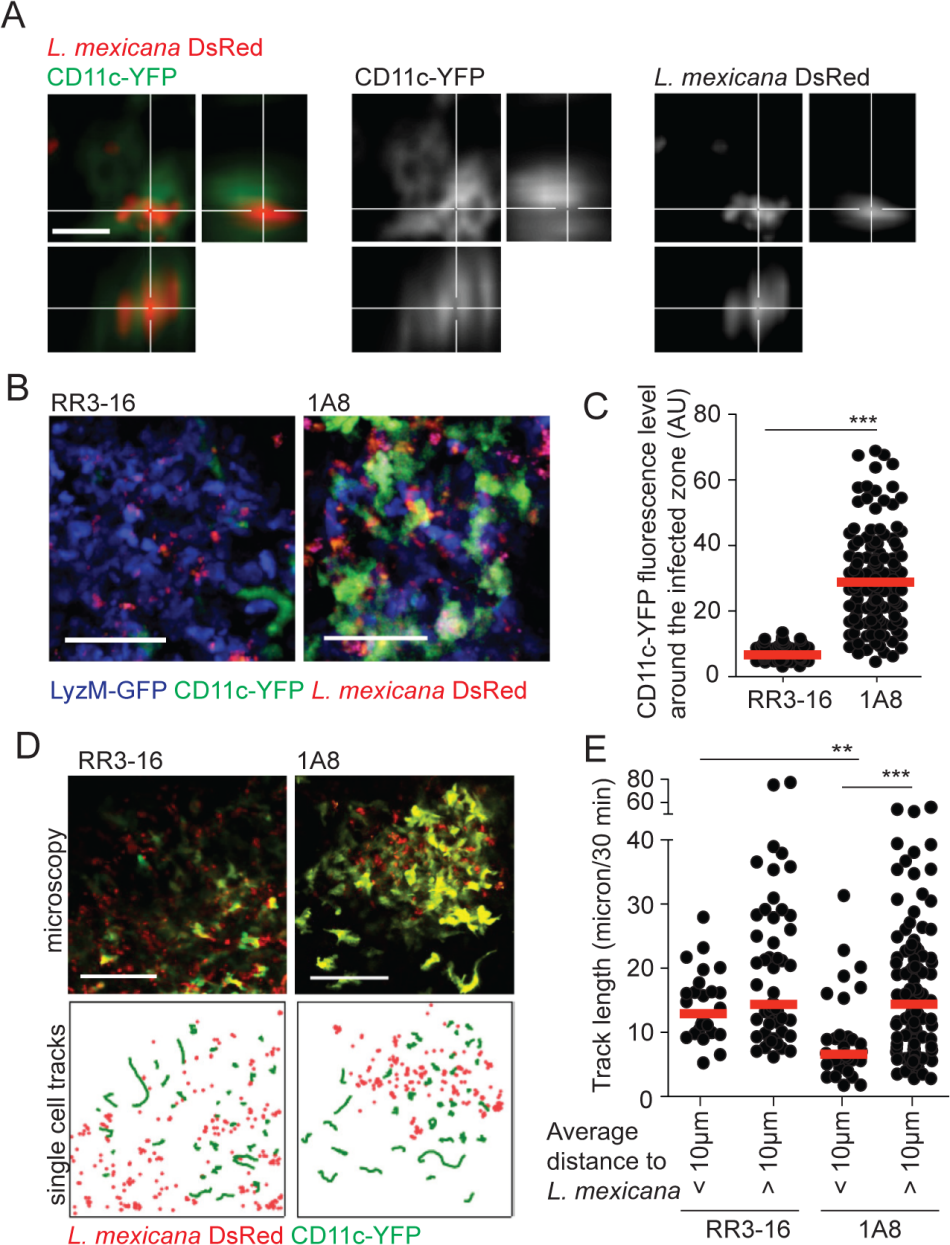
DC behavior at the site of infection in the absence of mature neutrophils. (A) *L*. *mexicana-*DsRed within CD11c^+^ dermal cells analyzed by intravital 2-photon imaging. A representative projection image across x, y and z dimensions obtained in a 1A8 PMN-depleted CD11c-YFP mouse infected for 24h with 10^6^ *L*. *mexicana* DsRed is shown, scale bar: 10 micron. (B) Representative intravital 2-photon imaging frames of ear dermis of CD11c-YFP/LyzM-GFP double transgenic mice infected for 24h with 10^6^ metacyclic *L*. *mexicana*-DsRed, RR3-16-treated (left) or 1A8 PMN-depleted (right). Scale bar: 50 micron. (C) Quantitative analysis of data shown in (B) representing YFP fluorescence in the 10 micron perimeter around *L*. *mexicana-*DsRed in presence or absence of neutrophils. Every dot represents the CD11c fluorescence levels around one *L*. *mexicana*-infected zone. (D) Representative intravital 2-photon imaging frames of ear dermis of CD11c-YFP transgenic mice 24 hours after infection with 10^6^ metacyclic *L*. *mexicana*-DsRed and injected either with RR3-16 control (left panels) or 1A8 PMN-depleting mAbs (right panels). A single frame z projection is shown (top). CD11c-YFP tracks were monitored over 30 minutes (green lines) and the positions of *L*. *mexicana*-DsRed at 15 minutes of imaging (red dots) are shown (bottom), scale bar: 100 micron. (E) CD11c-YFP cell tracks were grouped into cells entering a 10 micron perimeter of *L*. *mexicana*-DsRed and cells not entering this perimeter. Displacement over 30 minutes of intravital imaging was determined in RR3-16-treated and 1A8 PMN-depleted mice. Every dot represents one CD11c-YFP cell track, data were obtained from 3 z-projections of 20 micron per condition. Data presented as mean and representative of 3 independent experiments (n = 3). ** p<0.01 ***p<0.001.

To investigate the impact of early neutropenia on DC mobilization following parasite infection, LyzM-GFP/CD11c-YFP double transgenic mice depleted or not of neutrophils were inoculated with *L*. *mexicana* in the ear pinna and the site of infection was visualized 24 hours later by 2-photon microscopy. Although *L*. *mexicana* was phagocytosed by DCs in both control and neutropenic mice (Fig 5A), the DC distribution observed in the ear dermis of mice transiently depleted of neutrophils differed markedly from that of similarly infected control mice treated with the RR3-16 mAb. First, a higher frequency of DCs was observed at the site of infection in neutropenic compared to control mice (Fig 5B and S1 and S2 Movies). Second, the distribution of DCs at the site of infection was markedly changed as the YFP signal from DCs was mostly found in close proximity around the parasites in the infected zone of neutropenic mice (Fig 5C).

To better discriminate DC behavior at the site of infection, CD11c-YFP reporter mice were similarly infected and DC dynamics was analyzed by 2-photon imaging 24 hours later. The velocity of DCs located close (<10μm) but not that of DCs located further away from the parasites (>10μm) was markedly decreased in neutropenic mice. In contrast, DC mobility close and further from parasites did not differ in the ear dermis of control mice (Fig 5D and 5E, S3 and S4 Movies).

**Fig 6:**
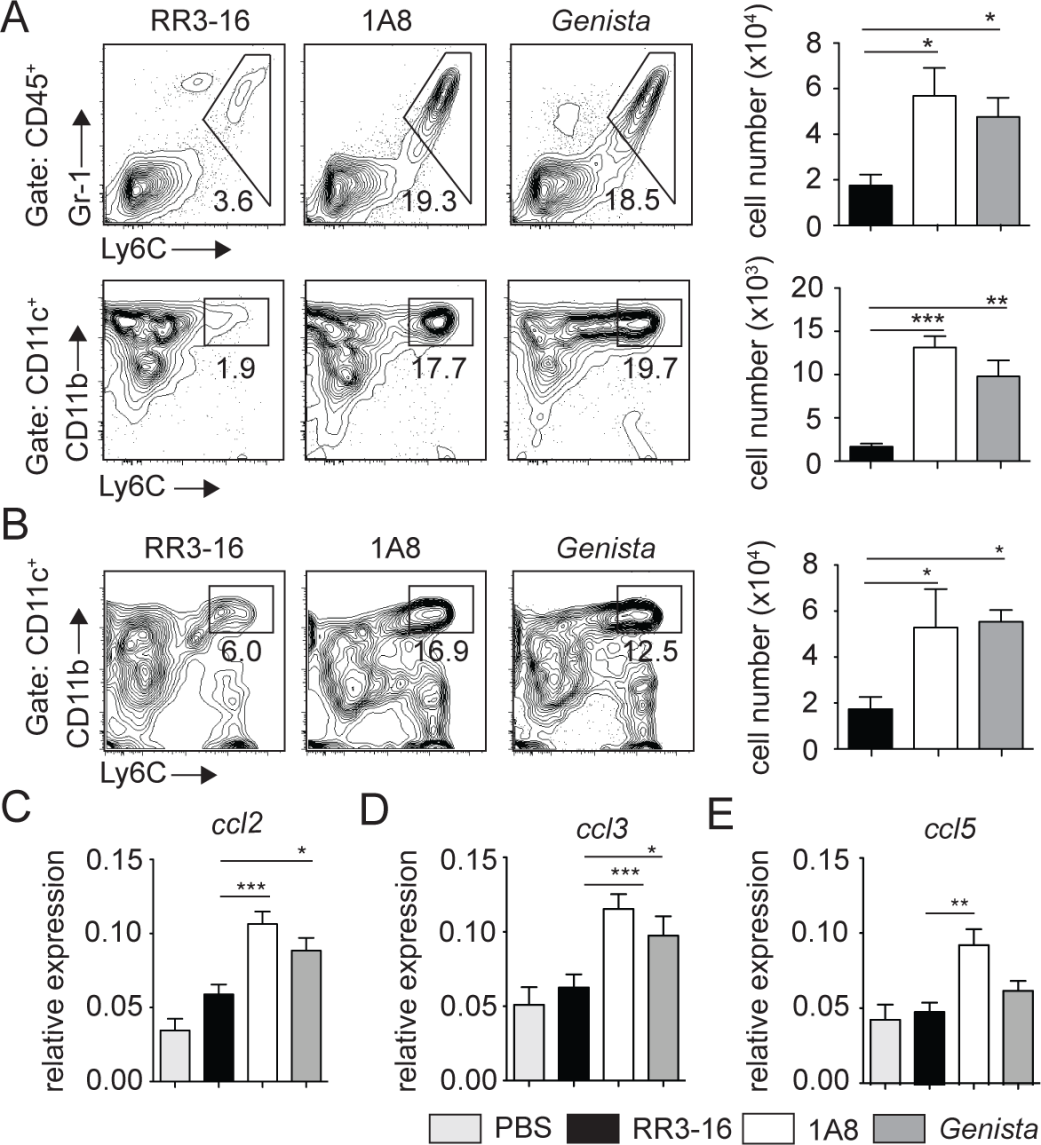
Enhanced recruitment of monocytes and MoDCs is observed in the absence of mature neutrophils early after infection. (A) RR3-16-treated, 1A8 PMN-depleted C57BL/6 and *Genista* mice were inoculated i.d. with 10^6^ metacyclic *L*. *mexicana*-DsRed. Ear and dLN cells were isolated 3 days following infection and the frequency of CD45^+^Gr1^+^Ly6C^+^ inflammatory monocytes (top) and CD45^+^CD11c^+^Ly6C^+^CD11b^+^ MoDCs (bottom) was analyzed by flow cytometry. Representative profiles are shown and the corresponding cell numbers given in the bar graphs. Data presented as the mean number of cells ± SEM (n = 6/group). (B) Representative flow cytometry profiles of dLN-derived CD45^+^CD11c^+^Ly6C^+^CD11b^+^ MoDCs in the three groups of mice described in (A). Quantitation in cell numbers is shown in the corresponding bar graphs. Data presented as the mean number of MoDCs ± SEM, (n = 6/group). (C-E) *Genista* mice, C57BL/6 mice depleted of neutrophils with the 1A8 mAb and C57BL/6 mice injected with the control RR3-16 mAb were infected with 10^6^ metacyclic *L*. *mexicana*. 72 hours post infection mRNA was isolated from the ear dermis. As controls, uninfected C57BL/6 mice were injected with PBS. The levels of (C) CCL2, (D) CCL3 and (E) CCL5 mRNA were analyzed by RT-PCR and normalized to those obtained for HPRT. Data are represented as relative expression. Shown is the mean ± SEM for n = 12/group. * p< 0.05 **p<0.01 *** p<0.001. The data are pooled from 3 independent experiments.

To determine if the increased frequency of dermal DCs observed by 2-photon intravital microscopy was sustained during the first days of infection, the ear dermis and draining lymph node (dLN) of mice inoculated with *L*. *mexicana* was analyzed 3 days p.i. Only poor recruitment of inflammatory monocytes and monocyte-derived DCs (MoDCs) was observed in C57BL/6 ear dermis in line with a previous report [5]. In contrast, a markedly increased frequency and number of inflammatory monocytes and MoDCs were recruited in the ear dermis of both PMN-depleted and *Genista* mice (Fig 6A). The frequency and number of MoDCs in the dLN were also increased (Fig 6B).

To investigate how neutrophils could affect monocyte and MoDC recruitment to the site of infection, mRNA levels of CCL2, CCL3 and CCL5, three chemokines involved in monocyte recruitment, were analyzed in the ears of *L*. *mexicana*-infected mice. 24 and 72 hours post infection, ears of *Genista*, RR3-16- or 1A8-treated C57BL/6 mice were isolated and chemokine mRNA levels analyzed. 24 hours post infection, all three chemokine mRNA levels were low with no observed increased mRNA levels in neutropenic mice (S4 Fig). 72 hours post infection chemokine mRNA levels remained low in infected C57BL/6 mice treated with the RR3-16 control mAb with values comparable to those of uninfected mice injected with PBS. In contrast, a significant increase in mRNA levels of the three chemokines was observed in neutropenic mice (Fig 6C–6E). Of note, among the three chemokines analyzed mRNA levels of *ccl2*, a key monocyte attractant, were the most induced. Collectively, these data suggest that the presence of *L*. *mexicana* within neutrophils impairs the transcription of monocyte-recruiting chemokines by cells present at the site of infection.

**Fig 7:**
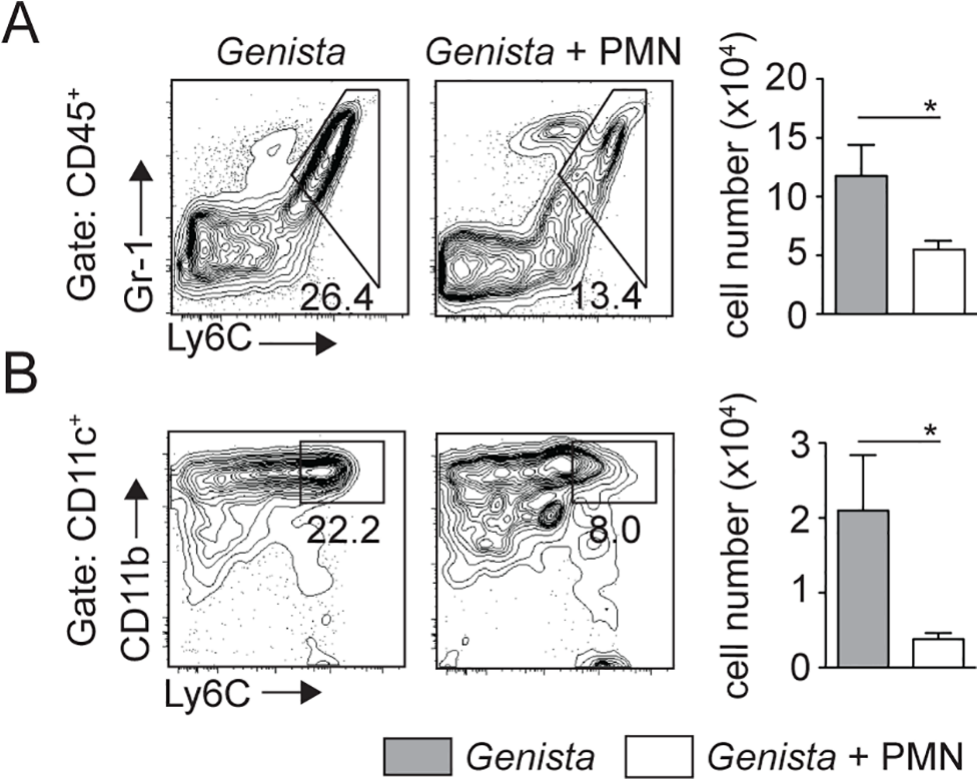
Injection of WT neutrophils in *Genista* mice reduces the otherwise observed monocyte recruitment at the site of *L*. *mexicana* inoculation. *Genista* mice were either inoculated i.d. with 10^6^ metacyclic *L*. *mexicana*-DsRed or co-inoculated i.d. with 10^6^ C57BL/6 sorted neutrophils. Ears were recovered 3 days post infection, processed and analyzed by flow cytometry. (A) Representative flow cytometry profiles of ear-derived CD45^+^Gr1^+^Ly6C^+^ inflammatory monocytes and of (B) CD45^+^CD11c^+^Ly6C^+^CD11b^+^ MoDCs are depicted with the corresponding quantitation in cell numbers shown in the bar graphs. Data presented as the mean number of cells ± SEM for n = 6/group. Results are representative of 4 or more independent experiments. * p<0.05.

To further demonstrate that the presence of neutrophils impaired the recruitment of dermal monocytes and MoDCs in C57BL/6 mice, 10^6^ sorted C57BL/6 neutrophils were adoptively transferred in the ear dermis of *Genista* mice at the time of *L*. *mexicana* inoculation. The presence of mature neutrophils in the *Genista* ear dermis markedly decreased the *L*. *mexicana-*induced recruitment of inflammatory monocytes and of MoDCs (Fig 7A and 7B). Collectively, these data show a striking negative effect of neutrophils on the recruitment and the behavior of DCs following infection with *L*. *mexicana*.

*Genista* and C57BL/6 mice depleted of neutrophils lack mature NK cells [24,26]. To address whether the increased monocyte and MoDC recruitment observed in neutropenic mice was based on a NK cell defect, C57BL/6 and neutropenic mice were depleted of NK cells prior to infection with *L*. *mexicana*. Depletion had no impact on monocyte and MoDC recruitment in both control and neutropenic mice (S5 Fig) ruling out a role for NK cells in the early recruitment of monocytes and MoDCs to the site of infection.

### Neutropenia favors the development of a protective immune response against *L*. *mexicana* infection

**Fig 8:**
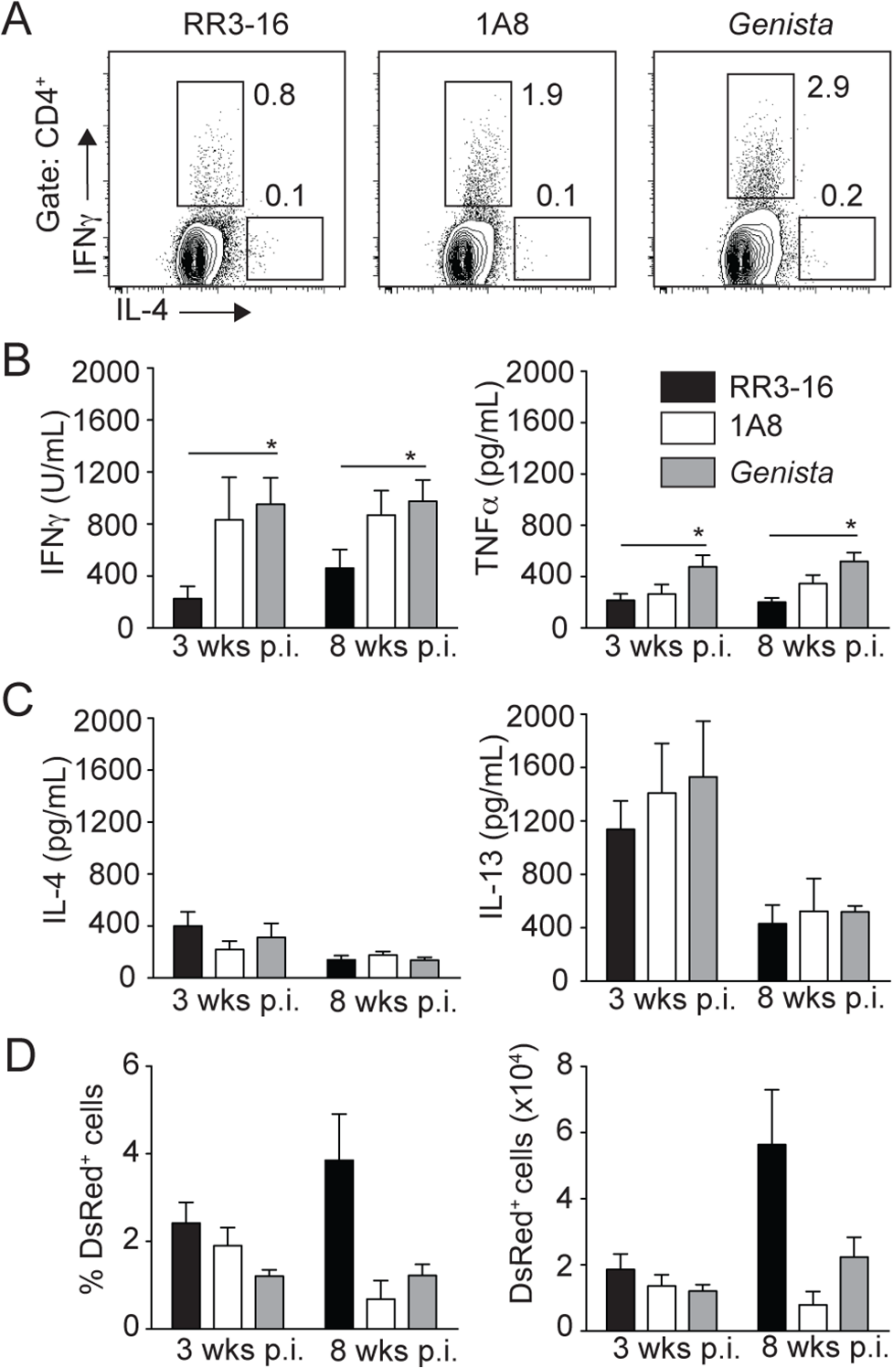
Early absence of neutrophils allows the development of *L*. *mexicana*-specific Th1 cells. (A) RR3-16-treated, 1A8 PMN-depleted C57BL/6 and *Genista* mice were inoculated i.d. with 10^6^ metacyclic *L*. *mexicana*-DsRed in the ear dermis. 3 and 8 weeks following infection, dLN cells were recovered and analyzed. Representative flow cytometry profiles of intracellular IL-4 and IFNγ expression in CD4^+^ dLN T cells isolated 3 weeks p.i. (B) 3 and 8 weeks following parasite inoculation secretion of IFNγ, TNFα (C) IL-4 and IL-13 was quantified by ELISA in supernatants of dLN cells restimulated with UV-irradiated parasites. Data presented as the mean cytokine secretion ± SEM, n = 4/group. (D) Frequency and number of parasitized dermal CD45^+^DsRed^+^ cells was analyzed by flow cytometry. Data presented as mean ± SEM for n ≥ 4/group. Data are representative of 2 independent experiments. * p<0.05.

To investigate the long-term impact of transient or sustained neutropenia on the development of *L*. *mexicana*-specific immune response, we analyzed IFNγ production in dLN cells of *Genista* and PMN-depleted mice as this cytokine induces macrophage-killing of *Leishmania* parasites. Three weeks post infection, neutropenic mice had a higher fraction of intracellular IFNγ^+^ CD4^+^ T cells in the dLN (Fig 8A). This corresponded to increased IFNγ secretion into the cell supernatant of parasite-restimulated dLN cells 3 and 8 weeks p.i. and TNFα secretion was also increased (Fig 8B). In contrast, Th2 cytokine levels such as IL-4 and IL-13 were not modified by neutropenia (Fig 8C). In line with the higher IFNγ levels observed in neutropenic mice, the frequency and number of infected cells were lower at 8 weeks post infection in these mice in the ear dermis (Fig 8D). Collectively, these data demonstrate that rapid sequestration of *L*. *mexicana* parasites by neutrophils severely limits the induction of an adaptive immune response, contributing to the development of chronic cutaneous disease.

## Discussion

Neutrophils are emerging as important players in the development of adaptive immunity. However little is known regarding their role in the development of chronic diseases, and there are currently contradictory findings on their role in the control of *Leishmania*. Here, we report and quantify for the first time *Leishmania*-induced NET formation shortly after parasite inoculation *in vivo*. Most microorganisms, including protozoan parasites such as *Toxoplasma gondii* [27] and *L*. *amazonensis* [21] are killed by NETs. In contrast, *L*. *mexicana*-induced NETs did not kill entrapped parasites. Similarly, *L*. *donovani*, *L*. *major* and *L*. *infantum* were reported to escape NET killing *in vitro*, a process associated with the presence of Lipophosphoglycan or 3’nucleotidase/nuclease, two molecules expressed in *Leishmania* promastigotes [22,28]. Thus evasion of NET killing by *Leishmania* promastigotes appears to be an important survival strategy in several *Leishmania* spp.

During natural sand fly infection an average of 5000 to 10 000 infectious promastigotes can be inoculated in the mouse host dermis [29], leading to massive local neutrophil recruitment which persists during the first days of infection [16]. However, needle inoculation of a relatively high dose (10^6^) of parasites was required to induce a comparable number of neutrophil recruitment in the ear pinna. The lack of neutrophil recruitment observed following needle inoculation of a low dose of parasites could be explained by the absence of vector-derived factors. Indeed, in addition to parasites, sand flies deliver salivary gland products [30, 31] together with a proteophosphoglycan rich gel of parasite origin named promastigote secretory gel (PSG) [32]. Both sand fly delivered factors contribute to the recruitment of inflammatory cells [31,33,34]. Furthermore, sand fly factors and PSG can exacerbate infection and interfere with the parasiticidal status of macrophages [33]. Interestingly, a sand fly salivary gland endonuclease from *Lutzomia Longipalpis*, the vector of *Leishmania infantum*, was recently shown to be able to digest NET scaffold *in vitro* [35]. It is not known which quantity of endonuclease or how long nuclease activity persists at the site of infection, but these data suggest that components present in salivary glands of some sand fly vectors could interfere with NET formation at least within the first hours of infection. It is therefore important to consider that salivary gland products and/or PSG contribute to neutrophil recruitment and may also influence early parasite survival shortly after infection, an area that would warrant further studies.

Susceptibility to *Leishmania* is complex and results from a combination of factors. Among these, *Leishmania* species express distinct virulence factors favoring their spreading within the host. Cysteine peptidase (CP) enzymes are important virulence factors present in parasites of the *L*. *mexicana* complex [36]. C57BL/6 mice infected with CPB-deficient *L*. *mexicana* can develop a Th1 immune response and heal their lesions [37]. Expression of *L*. *mexicana* CPB is stage specific, with the highest expression observed at the amastigote stage. Although *L*. *mexicana* promastigotes express less CPB than amastigotes, interactions between *L*. *mexicana* promastigote CPB and neutrophils could already have an impact on the onset of the immune response. CPB could contribute to the impaired secretion of chemokines and cytokines observed in response to *L*. *mexicana* and/or released CPB could modulate local cell recruitment through potential impact on chemokines, an effect previously reported by other protozoan CPBs [38,39]. Neutrophils with their numerous cytosolic proteases may contribute to CPB activity and favor chemokine inhibition, an area that remains to be investigated.

Distinct *Leishmania* species may have different impacts on neutrophils and the latters may play disease protective or susceptible roles, a process that can also be influenced by host genetic factors. *L*. *major* exposure elicited distinct innate receptors, cytokine and chemokine secretion in neutrophils from mice genetically resistant (C57BL/6) or susceptible (BALB/c) to infection [40,41]. Early neutrophil recruitment had a deleterious role on disease outcome in BALB/c mice and a transient protective role with no impact on disease outcome in C57BL/6 mice following *L*. *major* infection [15,42]. In contrast, a small transient protective role for neutrophils was observed following *L*. *amazonensis* infection in BALB/c but not C57BL/6 mice [43]. Here we show that sequestration of *L*. *mexicana* by neutrophils impairs the development of a protective response in C57BL/6 mice. We observed similar deleterious role for neutrophils in *L*. *mexicana* infected BALB/c mice (S6 Fig) suggesting that following *L*. *mexicana* infection, the negative impact of neutrophils on disease outcome results from parasite factors rather than from local host genetic factors.

In addition to genetic background, the dose and site of infection may also have an impact on disease outcome (reviewed in [3]). Thus during natural infection the influence of neutrophils on the various forms of the disease may depend on several factors including parasites and host genetic factors, the site of infection, and the number of parasites transmitted by the sand fly.

Several microorganisms use different strategies to inhibit neutrophil killing [20,44–47]. It was thus hypothesized that neutrophils could be used as a shuttle (Trojan horse) to enter into a host [48]. These data and those presented herein suggest that subversion of parasite uptake by neutrophils, which is observed at the very early phase of infection, may be used by several microorganisms to enhance initial pathogen survival and to prevent immune control.

In addition to their well-established role as the first line of defense against pathogens, neutrophils have recently emerged as important players in regulating the launching of the adaptive immune response, reviewed in [49,50]. Modulation of neutrophil apoptosis can delay the onset of the immune response, as observed following phagocytosis of *Mycobacterium tuberculosis* by neutrophils [25]. Interestingly, a similar delay in neutrophil apoptosis was reported *in vitro* following *L*. *major* phagocytosis by human blood or mouse inflammatory neutrophils [20,51–53]. In contrast 80–90% of mouse parasitized dermal neutrophils were apoptotic 12 hours after needle inoculation of *L*. *major* i.d., contributing to neutrophil efferocytosis by DCs [19]. These differences could be due to the origin of the neutrophils used in these studies. Here, 24 hours after *L*. *mexicana* inoculation at a time when parasite presence is at its highest, parasitized dermal neutrophils did not show increased apoptosis compared to non-parasitized neutrophils, suggesting that *L*. *major* and *L*. *mexicana* use distinct strategies within neutrophils to interfere with the launching of a protective immune response.

During the first weeks following *L*. *mexicana* but not *L*. *major* inoculation, only poor local recruitment of inflammatory monocytes and MoDCs was observed in agreement with a previous study [5]. We found enhanced and rapid recruitment of monocytes and MoDCs to the site of parasite inoculation in neutropenic mice and a reversal of this effect when wild type neutrophils were adoptively transferred into *Genista* mice. In neutropenic mice, the increased number of monocytes observed after *L*. *mexicana* infection was correlated with an increased frequency of MoDCs. Neutrophils were recently reported to contribute to NK cell activation [26] in line with the predominant presence of immature neutrophils in GAPDH patients and *Genista* mice at homeostasis [24]. Depletion of NK cells did not modify the high inflammatory monocyte and MoDC recruitment observed in neutropenic mice, suggesting that the low number of NK cells recruited in the ear dermis early after infection does not contribute to the observed phenotype.

Increasing evidence suggests crosstalks between neutrophils and DCs resulting in enhanced DC recruitment, activation or inhibition depending on the context, reviewed in [54]. Several mechanisms may explain why sequestration of *L*. *mexicana*, but not *L*. *major* by neutrophils impairs early recruitment of monocytes and MoDCs. Chemokines are playing important roles in the onset of immune response against *Leishmania* infection [55]. *L*. *major*-induced neutrophil secretion of CCL3 leads to the recruitment of inflammatory monocytes and MoDCs [41], a process which plays a critical role in the priming of a Th1 response to *L*. *major* infection [56]. Following *L*. *mexicana* infection, we show here that 3 days post infection *ccl3* mRNAs is induced at the site of infection selectively in neutropenic mice. CCL3 may thus also contribute to the development of a protective immune response following *L*. *mexicana* infection in neutropenic mice. However, unlike what was previously reported for *L*. *major*, the source of this chemokine originates from other cells than neutrophils. Moreover, *ccl2* and *ccl5 mRNA* levels were also induced specifically in the ear dermis of neutropenic mice. Interestingly, in addition to its role in recruiting monocytes and DCs, CCL2 was also shown to synergize with IFN-γ to activate macrophage microbicidal activity [57] and thereby the presence of this chemokine could further contribute to healing. Collectively, we show here that trapping of *L*. *mexicana* by neutrophils impairs the induction of monocyte and DC-attracting chemokine mRNAs at the site of infection, correlating with the poor monocyte and MoDC recruitment observed in response to this parasite 3 days post infection, and with the subsequent impaired Th1 cell differentiation.

Using 2-photon microscopy we further showed that parasite sequestration by neutrophils not only impaired DC migration to the site of infection but also their mobility and interactions with parasites. Parasites were phagocytosed by DCs in both neutropenic and control mice. However, considering the higher frequency of DCs recruited locally in neutropenic mice, the frequency of parasitized DCs is likely to be significantly higher in these mice. As DCs are crucial for launching adaptive immunity, our results reveal that antigen presentation and priming of the adaptive immune response is improved in the absence of neutrophils. Interestingly, the lesion size in neutropenic mice started to diminish shortly after the surge in IFNγ secretion in dLN cells. Full lesion resolution correlated with high IFNγ secretion and low IL-13 and IL-4 levels in dLN cells of neutropenic mice, suggesting an important role for IFNγ in this process. It is noteworthy that lesion size resolution was observed in both *Genista* and in mice only transiently depleted of neutrophils at the onset of infection. The latter demonstrates that neutrophils determine long-term disease progression by impacting events occurring during the first hours of infection.

Several studies reported that neutrophils can migrate transiently to the dLN after infection, injection of adjuvants or vaccination [15,58–62]. Following *L*. *mexicana* infection most of the impact on the immune response appears to result from early sequestration of parasite by neutrophils. Nevertheless, one cannot exclude that a low number of neutrophils that migrate to the dLN early in infection could also contribute to the impaired development of a protective immune response.

Collectively, our findings show that *L*. *mexicana* use neutrophils as a safe transient shelter. In addition, parasite presence within these cells or in association with NETs fails to recruit immune cells that are essential for the launching of an effective parasite-specific immune response and resolution of lesion size. These data reveal an unexpected role for neutrophils to promote chronic diseases suggesting that strategies to modify neutrophil behavior will improve the development of protective immune responses against *Leishmania* infection.

## Materials and Methods

### Mice

C57BL/6 and BALB/c mice were purchased from Charles Rivers. *Genista* [24], LyzM-GFP [63] a gift from Prof. Sussan Nourshargh (Queen Mary, University of London), CD11c-YFP [64] a gift from Prof. Cornelia Halin (ETH, Zürich) and LyzM-GFP/CD11c-YFP mice were bred under pathogen-free conditions at the Epalinges Center. 5–10 weeks old females were used.

### Ethics statement

All animal experimental protocols were approved by the veterinary office regulations of the State of Vaud, Switzerland, authorization 1266.4–6 to FTC and performed in compliance with Swiss laws for animal protection.

### Parasites and infections

*L*. *mexicana* (MYNC/BZ/62/M379) WT,-DsRed (gift of Prof. Tony Aebischer, Robert Koch-Institut, Berlin) and-luciferase expressing parasites were cultured at 26°C in complete M199 medium (10% fetal bovine serum, 4% HEPES, 1% PNS). Transgenic *L*. *mexicana* were cultured with hygromycin B (Sigma) at 50μg/mL. For infections, 1 x 10^6^ metacyclic parasites were needle injected into the ear dermis in iDMEM in a final volume of 10μL. In PMN-parasites co-injection experiments, 10^6^ negatively MACS-sorted BM-PMN (Miltenyi Biotec) and 10^6^ metacyclic parasites were co-injected in a final volume of 20μL. For long-term experiments, lesion length and width were weekly measured using an electronic Vernier caliper and lesion score was calculated as described previously [65].

### mRNA isolation and real-time PCR

Ears were harvested at the indicated times after infection, homogenized using a tissue lyser (Qiagen, Hildren, Germany) and mRNA was extracted as previously described [66]. cDNA was prepared as described previously [66] and quantitative real-time PCR was performed using SYBR green on a LightCycler system (Roche). Primers for CCL2 (5’-TTAAAAACCTGGATCGGAACCAA-3’, 5’-GCATTAGCTTCAGATTTACGGGT-3’; GenBank Accession NM_01333), were used, and CCL3 and CCL5 were previously described [53]. The results were normalized to the hypoxanthine phosphoribosyltransferase (HPRT) housekeeping gene using the comparative threshold cycle method (CT) for relative quantification.

### Generation of luciferase-expressing *Leishmania mexicana* transgenic cell lines

pGL1313 was linearized by Pme1 and Pacl restriction digestion, purified and used for electroporation using the Amaxa nucleofaector system as previously described [67]. Briefly, 7 x 10^6^ *L*. *mexicana* promastigotes at logarithmic growth phase were harvested and resuspended in 100 μl Human T Cell Nucleofector Solution. The transfected cells were cloned by serial dilution in presence of 50 μg/ml of hygromycin B (Sigma). Clones resistant to the antibiotic were obtained and Luciferase expression validated by the determination of luciferase activity.

### Neutrophil depletion

Six hours before and thirty-six hours after parasite inoculation, mice were given i.p. 150μg of the 1A8 mAb (BioXcell) that selectively binds to mouse neutrophils [68]. As a control, mice were given i.p. 150μg of the RR3-16 mock mAb directed against the Vα3.2 chain of the T cell receptor (gift from R. MacDonald, Ludwig Institute/UNIL, Epalinges, Switzerland).

### Isolation of dLN and ear mouse cells

Infected ears and dLNs were isolated and processed to single cell suspensions. Briefly, ears were recovered, homogenized in iDMEM containing 0.2 mg/ml Liberase TL (Roche) for 2h at 37°C and then filtered using 40μm filters (Falcon). dLN cells were isolated and stained for flow cytometry analysis. For intracellular cytokine staining, 1 x 10^6^ dLN cells were stimulated with 50μg/mL PMA, 500μg/mL Ionomycin and 1μg/mL Brefeldin A for 4h. Live parasites were determined by limiting dilution assay as described previously [15].

### Cytokine production

10^6^ dLN cells were cultured ± UV-irradiated *L*. *mexicana* promastigotes (MOI of 1:5) for 72h at 37°C. Supernatants were recovered and cytokine production analyzed by ELISA following manufacturer’s instructions. (BD Biosciences; R&D). IFNγ secretion was detected using a homemade ELISA kit as previously described [15].

### Flow cytometry and MPO luminescence

Stained cells were analyzed using a BD LSR II-SORP system (Becton Dickinson) and analyzed with FlowJo software (Tree Star). The following mAbs were used: CD45-PerCPCy5, CD45-APC, CD8-APC, anti-IFNγ-PECy7 and anti-IL-4-FITC (BD Biosciences), Gr1-APC, CD11c-PECy7, CD11b-PB, F4/80-APC (eBiosciences), 1A8-APCCy7, Ly6C-FITC, 1A8-APCCy7, CD4-AF700. DAPI and AnV-FITC (BioLegends). *In vivo* bioluminescence imaging of MPO activity was quantified through injection of 200mg/kg luminol (Carbosynth) i.p. 10 minutes before luminescence acquisition. Photon emission was acquired for 10 minutes using a Xenogen IVIS Imaging system.

### Intravital 2-photon microscopy

Mice were anaesthetized with 80mg/kg Ketamin and 8mg/kg Rompun. The ear pinna was spread with the inner side on top on a custom made stage heated to 37°C and processed as reported previously [69]. Intravital imaging was performed using a Zeiss LSM 710 NLO microscope equipped with a Cameleon Ultra II Ti:Sapphire 2-Photon laser (Coherent) tuned at 960 nm. Emitted fluorescence during concomitant GFP, YFP and DsRed imaging was read out using the descanned detectors of the microscope imaging at 500–520 nm, 525–555 nm, and 560–650 nm, respectively. Two-color YFP and DsRed imaging was detected with non-descanned detectors at 510–555 nm and 560–650 nm, respectively. Four-dimensional full-scale images were analyzed using the Tracking and Colocalization modules of the Imaris Software (Bitplane, Zurich, Switzerland) and the ImageJ software. Tracking of DCs was performed on 20μm Z-projections, distances to the closest parasite were determined automatically for every DC position of the track.

### Detection of NETs

3 x 10^5^ negatively selected MACS-sorted (Miltenyi Biotec) mouse BM-neutrophils were seeded on poly-L-lysine-coated coverslips (BD Biosciences) for 30 min at 37°C with *L*. *mexicana*-DsRed at a MOI of 5:1 (parasite:cell) or with PMA or LPS at 100ng/mL with or without 10μg/mL DNase for 4h at 37°C. Neutrophil purity was >95%. Cells were fixed with 4% PFA (Sigma) and stained using polyclonal Rabbit anti-Human MPO (Dako) and Alexa Fluor 488 secondary mAbs (Life Technologies). Coverslips mounted in DAPI mounting medium (Life Technologies) were analyzed by confocal laser scanning microscopy (LSM 510 META; Carl Zeiss). For NET killing assay, 10^5^ neutrophils were incubated with *L*. *mexicana*-luciferase at a parasite-cell MOI of 5:1 with or without DNase as described above. Parasites treated or not with DNase were used as controls. Luciferase activity was measured using the luciferase assay system (Promega) and quantified using a MicroLumatPlus luminometer (EG&G Berthold).

NETs presence in ear pinna sections was identified as previously described [70,71],[72] on ears recovered 24 hours following infection. Briefly, immunofluorescence analyses were performed on 4-mm formaldehyde-fixed and paraffin-embedded mouse ear sections using polyclonal anti-MPO antibody (Santa Cruz Biotechnology) and appropriate secondary Abs labeled with Alexa Fluor 488 (Life Technologies). Propidium iodide (Life Technologies) was used for DNA detection. Specimens were mounted in fluorescence mounting medium (Life Technologies) and analyzed by confocal laser scanning microscopy (LSM 5 EXCITER; Carl Zeiss). Total infiltration neutrophils and DNA-releasing neutrophils were counted in 10 consecutive fields for each specimen.

### Statistics

All p values were determined with Prism software (GraphPad Software Inc.) using the Student’s t-test for unpaired data. 2-photon imaging data was analysed with a Kruskal-Wallis test with a Dunn’s post test. The degree of significance were indicated as: * p<0.05, **p<0.01, ***p<0.001.

## Supporting Information

S1 Fig *L*. *mexicana* dose dependent neutrophil recruitment. (A) C57BL/6 mice were i.d. inoculated with 10^3^, 10^4^, 10^5^ or 10^6^ metacyclic *L*. *mexicana* parasites. Ear cells were isolated 24 hours post infection and CD45^+^Gr1^+^Ly6C^int^ neutrophil recruitment was analyzed by flow cytometry. Representative dot plots are shown, as well as quantitation of (B) neutrophil frequency and (C) number. Data presented as mean ± SEM. Data shown are representative of >2 independent experiments for n = 4. * p<0.05.
(TIF)

S2 Fig Neutrophil recruitment due to needle inoculation. (A) Neutrophil recruitment in the ear dermis of C57BL/6 mice inoculated with PBS. Representative flow cytometry profiles of ear-derived neutrophils and (B) chemiluminescent ear signals (MPO activity) after i.p. delivery of luminol at the indicated times after infection.
(TIF)

S3 Fig Neutrophil depletion efficacy using 1A8 mAb. LyzM-GFP mice were inoculated i.d. with 10^6^ metacyclic *L*. *mexicana* WT. 12 hours post infection, ear cells were isolated and blood taken and stained with the Ly6G mAb. Representative flow cytometry profiles in RR3-treated versus 1A8 PMN-depleted mice are shown, with neutrophils as LyzM/Ly6G double positive cells. Data representative of 2 independent experiments for n = 4.
(TIF)

S4 Fig Expression of *ccl3*, *ccl4* and *ccl5* mRNA in the ear dermis of mice 24 hours post *L*. *mexicana* infection. *Genista* mice, C57BL/6 mice depleted of neutrophils with the 1A8 mAb and C57BL/6 mice injected with the control RR3-16 mAb were infected with 10^6^ metacyclic *L*. *mexicana*. 24 hours post infection mRNA was isolated from the ear dermis. The levels of (A) CCL2, (B) CCL3 and (C) CCL5 mRNA were analyzed by RT-PCR and normalized to those obtained for HPRT. Data are represented as relative expression. Shown is the mean ± SEM for n = 4/group. * p< 0.05. The data are representative of 2 experiments.
(TIFF)

S5 Fig NK cells do not affect ear cellular recruitment 3 days following infection. (A) Ear NK cell recruitment kinetics in RR3-treated, 1A8 PMN-depleted and *Genista* mice following i.d. infection of 10^6^ metacyclic *L*. *mexicana* WT. Data shown as mean percentage ± SEM (left) and mean cell number ± SEM (right). (B) RR3-16-treated, 1A8-treated (PMN-depleted), NK1.1-treated (NK-depleted), 1A8/NK1.1-treated (PMN and NK co-depleted) C57BL/6 mice were inoculated i.d. with 10^6^ metacyclic *L*. *mexicana* WT. RR3-16 and 1A8 mAbs were injected as previously described, NK1.1 mAb was injected at a dose of 400μg i.v. 24 hours prior to infection. Ear cells were isolated 3 days following infection and the frequency of CD45^+^Gr1^+^Ly6C^+^ inflammatory monocytes and (C) CD45^+^CD11c^+^Ly6C^+^CD11b^+^ MoDCs were analyzed by flow cytometry. Data presented as mean percentage of cells ± SEM (left panels) and mean cell number ± SEM (right panels). Data shown are representative of ≥2 independent experiments for n = 6. * p<0.05. **p<0.01 *** p<0.001.
(TIF)

S6 Fig Transient neutropenia in BALB/c mice infected with *L*. *mexicana* results in lesion healing. RR3-16-treated and 1A8 PMN-depleted BALB/c mice were inoculated i.d. with 10^6^ metacyclic *L*. *mexicana*-DsRed parasites. Ear cells were isolated 3 days following infection and the frequency of (A) CD45^+^Gr1^+^Ly6C^int^ neutrophils (B) CD45^+^Gr1^+^Ly6C^+^ inflammatory monocytes and (C) CD45^+^CD11c^+^Ly6C^+^CD11b^+^ MoDCs was analyzed by flow cytometry. Quantitation in frequency is given and presented as the mean ± SEM (n = 4/group). (D) Impact of early neutropenia on lesion development following i.d. inoculation of 10^6^ metacyclic *L*. *mexicana* in BALB/c mice. BALB/c mice were treated with the 1A8 PMN-depleting mAb resulting in neutropenia during the first three days of infection or with the control RR3-16 mAb. Ear lesion scores were measured on a weekly basis as described in the *Materials and Methods*. Results are presented as mean lesion score ± SEM * p<0.05 ** p<0.005.
(TIF)

S1 Movie Dynamics in the ear of a RR3-16-treated LyzM-GFP/CD11c-YFP mouse 24h p.i. A RR3-16-treated LyzM-GFP/CD11c-YFP mouse was inoculated i.d. with 10^6^ metacyclic *L*. *mexicana* DsRed in a volume of 5–10μL. 24 hours after infection, the mouse was anesthetized and 2-photon imaging of the ear pinna was performed as described in the *Material and Methods*. Emitted fluorescence was read out using the descanned detectors of the microscope imaging at 500–520 nm, 525–555 nm, and 560–650 nm. A 28 minutes representative movie of 4 is shown. Scale bar: 20 micron.
(MOV)

S2 Movie Dynamics in the ear of a 1A8 PMN-depleted LyzM-GFP/CD11c-YFP mouse 24h p.i. A 1A8 PMN-depleted LyzM-GFP/CD11c-YFP mouse was inoculated i.d. with 10^6^ metacyclic *L*. *mexicana* DsRed in a volume of 5–10μL. 24 hours after infection, the mouse was anesthetized and 2-photon imaging of the ear pinna was performed as described in the *Material and Methods*. Emitted fluorescence was read out using the descanned detectors of the microscope imaging at 500–520 nm, 525–555 nm, and 560–650 nm. A 28 minutes representative movie of 4 is shown. Scale bar: 20 micron.
(MOV)

S3 Movie Dynamics in the ear of a RR3-16-treated CD11c-YFP mouse 24h p.i. A RR3-16-treated CD11c-YFP mouse was inoculated i.d. with 10^6^ metacyclic *L*. *mexicana* DsRed in a volume of 5–10μL. 24 hours after infection, the mouse was anesthetized and 2-photon imaging of the ear pinna was performed as described in the *Material and Methods*. Emitted fluorescence was read out using the non-descanned detectors at 510–555 nm and 560–650 nm. A 29 minutes representative movie of 4 is shown. Scale bar: 20 micron.
(MOV)

S4 Movie Dynamics in the ear of a 1A8 PMN-depleted CD11c-YFP mouse 24h p.i. A 1A8 PMN-depleted CD11c-YFP mouse was inoculated i.d. with 10^6^ metacyclic *L*. *mexicana* DsRed in a volume of 5–10μL. 24 hours after infection, the mouse was anesthetized and 2-photon imaging of the ear pinna was performed as described in the *Material and Methods*. Emitted fluorescence was read out using the non-descanned detectors at 510–555 nm and 560–650 nm. A 29 minutes representative movie of 4 is shown. Scale bar: 20 micron.
(MOV)
