# Supplementary figures and images for: Rapid Sequestration of Leishmania mexicana by Neutrophils Contributes to the Development of Chronic Lesion
Source: PLoS Pathog. 2015 May 28;11(5):e1004929. doi: 10.1371/journal.ppat.1004929 (PMC4447405; doi:10.1371/journal.ppat.1004929)

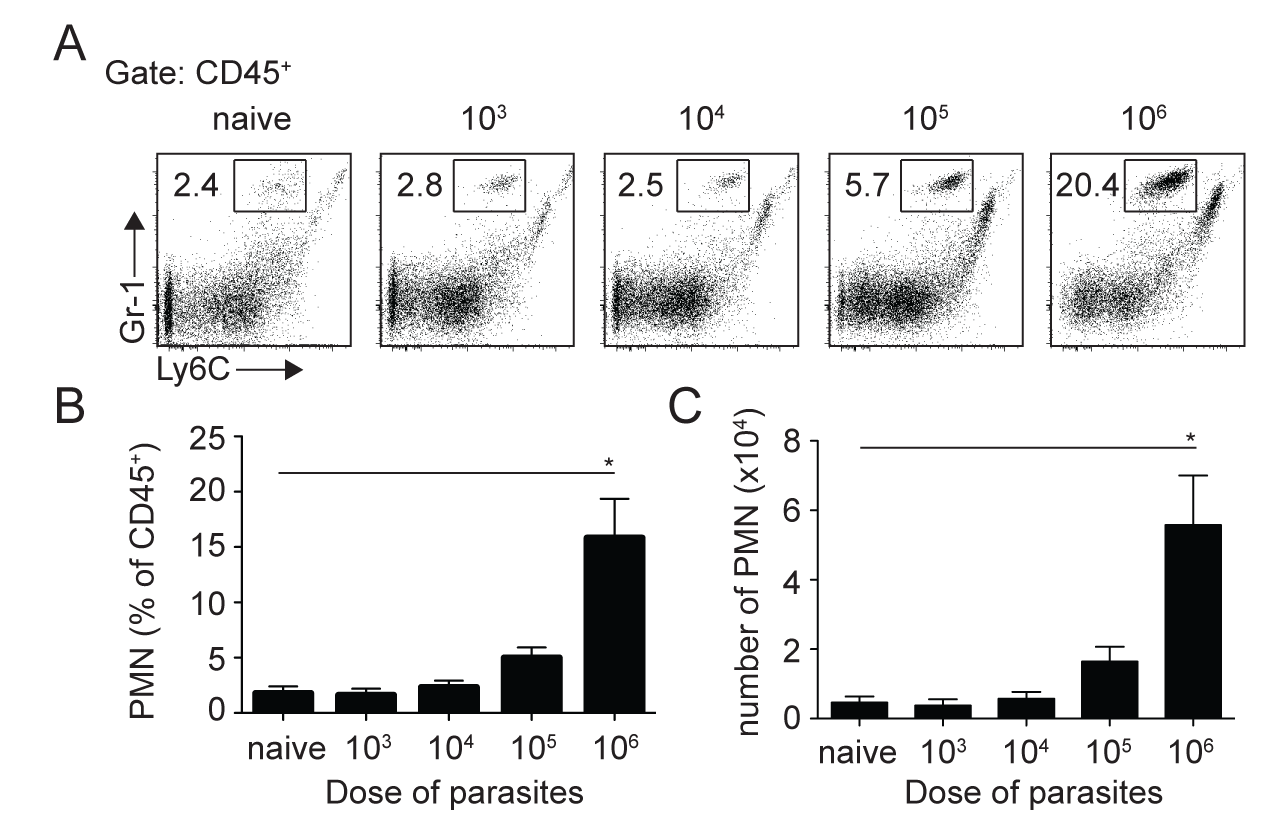

Supplement: S1 Fig — (A) C57BL/6 mice were i.d. inoculated with 103, 104, 105 or 106 metacyclic L. mexicana parasites. Ear cells were isolated 24 hours post infection and CD45+Gr1+Ly6Cint neutrophil recruitment was analyzed by flow cytometry. Representative dot plots are shown, as well as quantitation of (B) neutrophil frequency and (C) number. Data presented as mean ± SEM. Data shown are representative of >2 independent experiments for n = 4. * p<0.05. (TIF) [file ppat.1004929.s001.tif]

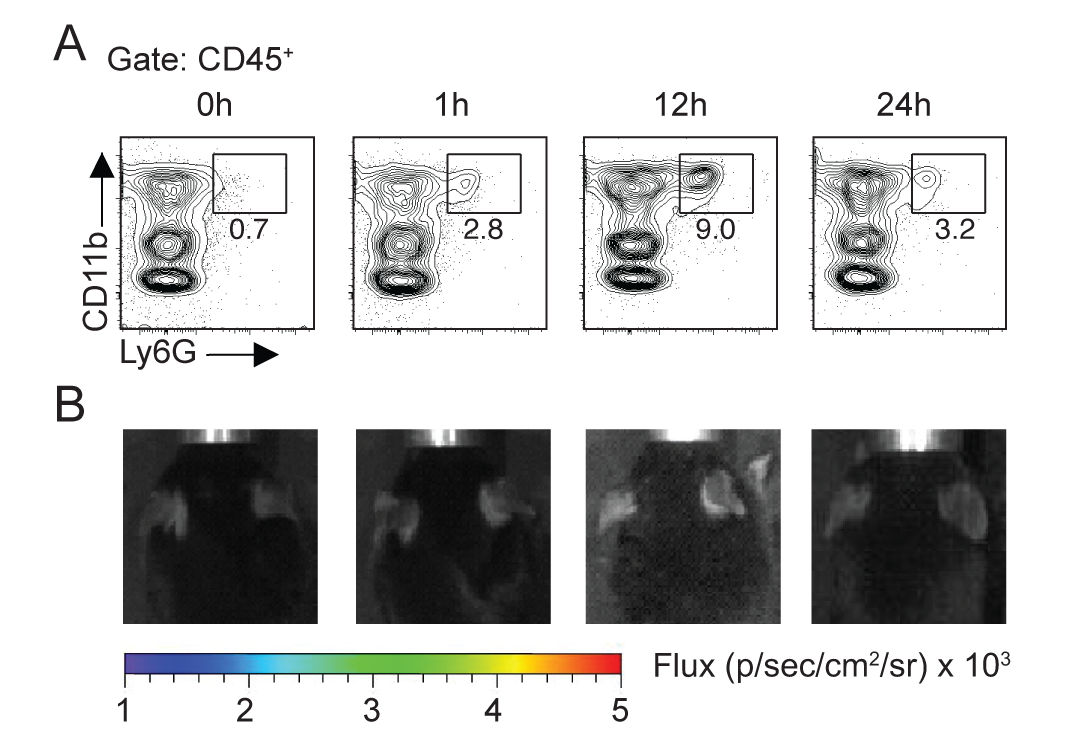

Supplement: S2 Fig — (A) Neutrophil recruitment in the ear dermis of C57BL/6 mice inoculated with PBS. Representative flow cytometry profiles of ear-derived neutrophils and (B) chemiluminescent ear signals (MPO activity) after i.p. delivery of luminol at the indicated times after infection. (TIF) [file ppat.1004929.s002.tif]

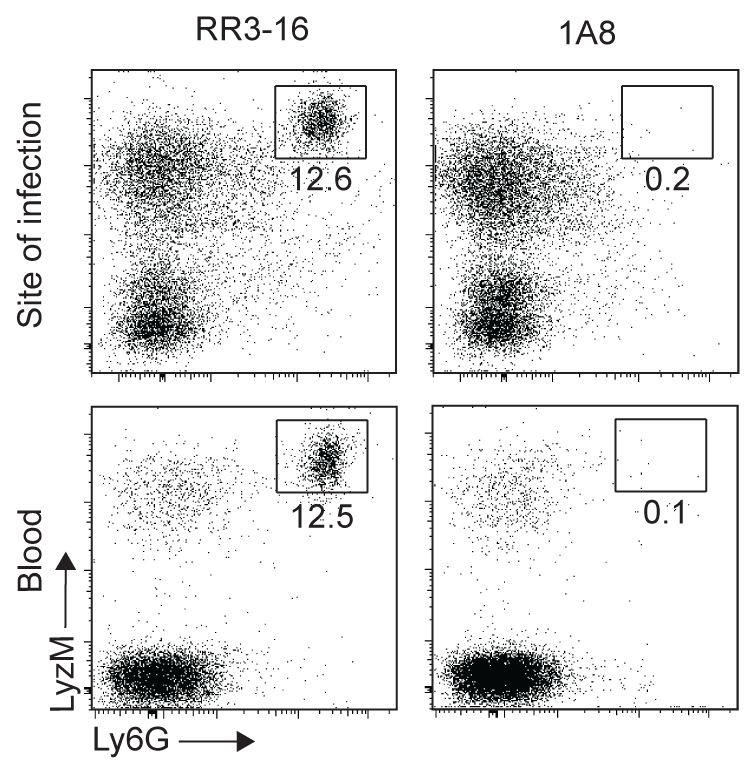

Supplement: S3 Fig — LyzM-GFP mice were inoculated i.d. with 106 metacyclic L. mexicana WT. 12 hours post infection, ear cells were isolated and blood taken and stained with the Ly6G mAb. Representative flow cytometry profiles in RR3-treated versus 1A8 PMN-depleted mice are shown, with neutrophils as LyzM/Ly6G double positive cells. Data representative of 2 independent experiments for n = 4. (TIF) [file ppat.1004929.s003.tif]

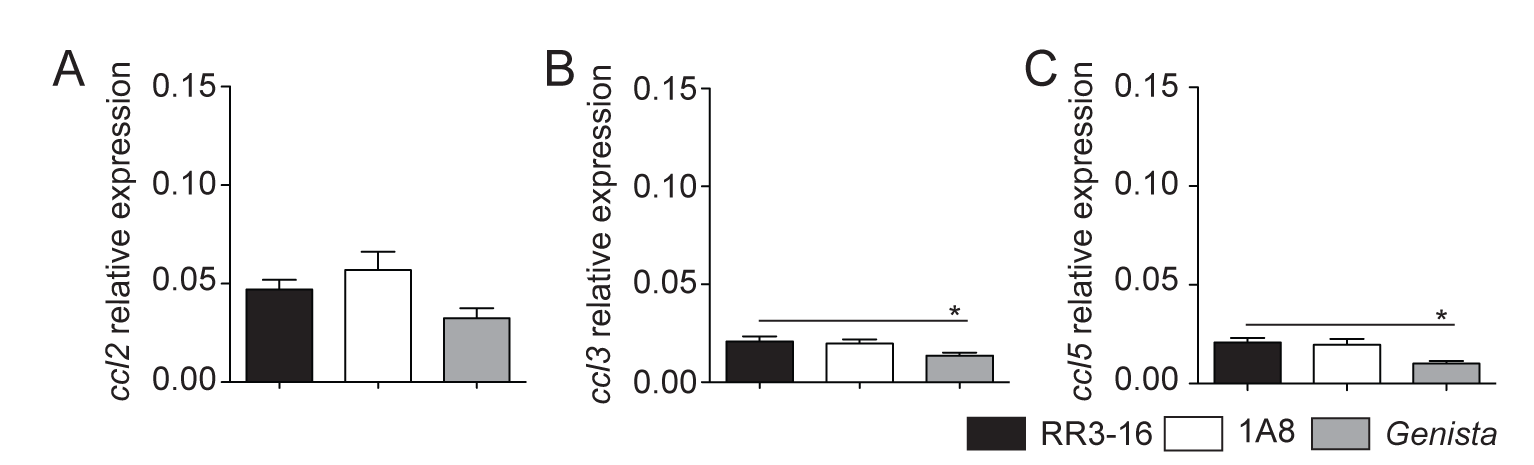

Supplement: S4 Fig — Genista mice, C57BL/6 mice depleted of neutrophils with the 1A8 mAb and C57BL/6 mice injected with the control RR3-16 mAb were infected with 106 metacyclic L. mexicana. 24 hours post infection mRNA was isolated from the ear dermis. The levels of (A) CCL2, (B) CCL3 and (C) CCL5 mRNA were analyzed by RT-PCR and normalized to those obtained for HPRT. Data are represented as relative expression. Shown is the mean ± SEM for n = 4/group. * p< 0.05. The data are representative of 2 experiments. (TIFF) [file ppat.1004929.s004.tiff]

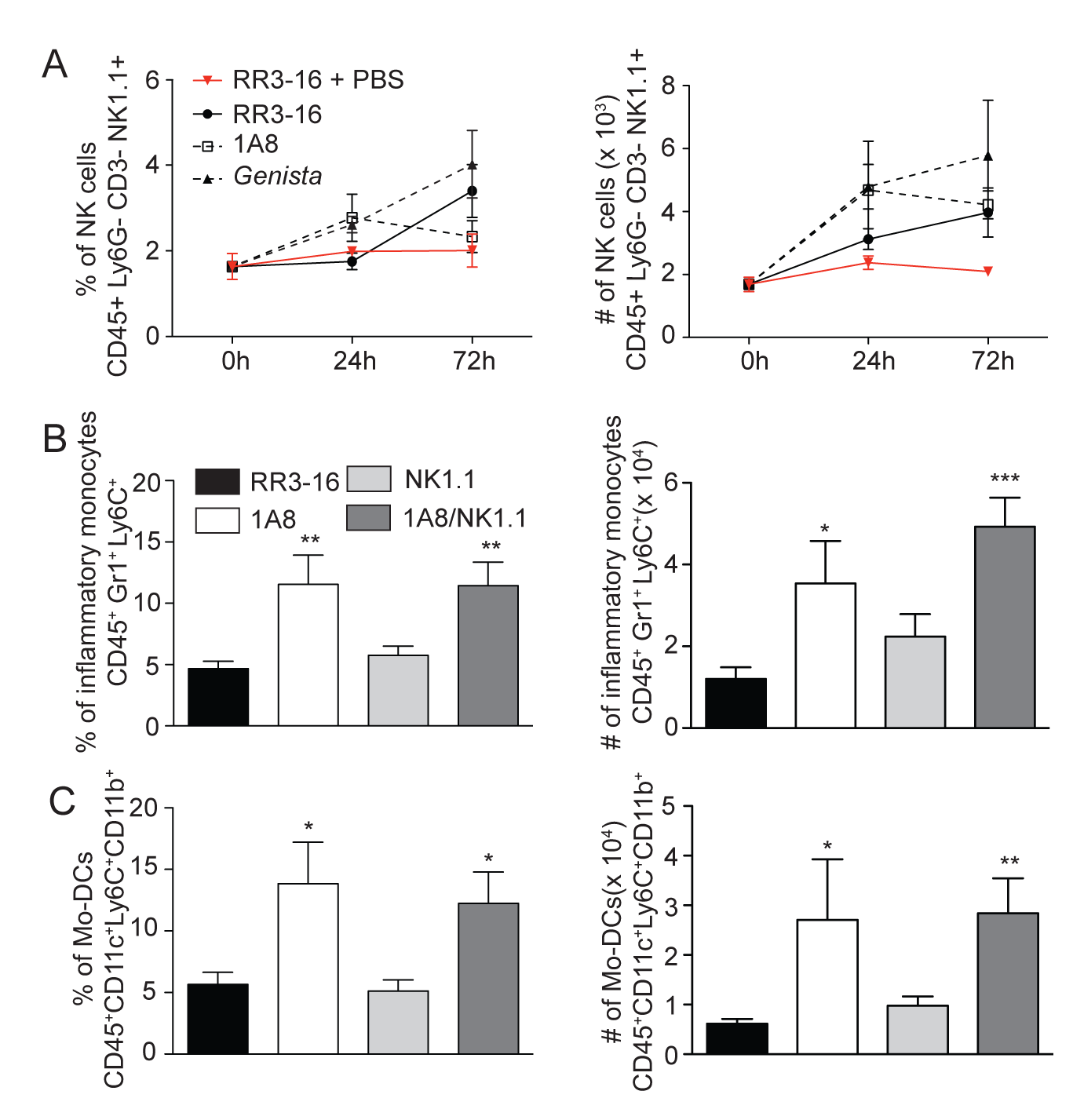

Supplement: S5 Fig — (A) Ear NK cell recruitment kinetics in RR3-treated, 1A8 PMN-depleted and Genista mice following i.d. infection of 106 metacyclic L. mexicana WT. Data shown as mean percentage ± SEM (left) and mean cell number ± SEM (right). (B) RR3-16-treated, 1A8-treated (PMN-depleted), NK1.1-treated (NK-depleted), 1A8/NK1.1-treated (PMN and NK co-depleted) C57BL/6 mice were inoculated i.d. with 106 metacyclic L. mexicana WT. RR3-16 and 1A8 mAbs were injected as previously described, NK1.1 mAb was injected at a dose of 400μg i.v. 24 hours prior to infection. Ear cells were isolated 3 days following infection and the frequency of CD45+Gr1+Ly6C+ inflammatory monocytes and (C) CD45+CD11c+Ly6C+CD11b+ MoDCs were analyzed by flow cytometry. Data presented as mean percentage of cells ± SEM (left panels) and mean cell number ± SEM (right panels). Data shown are representative of ≥2 independent experiments for n = 6. * p<0.05. **p<0.01 *** p<0.001. (TIF) [file ppat.1004929.s005.tif]

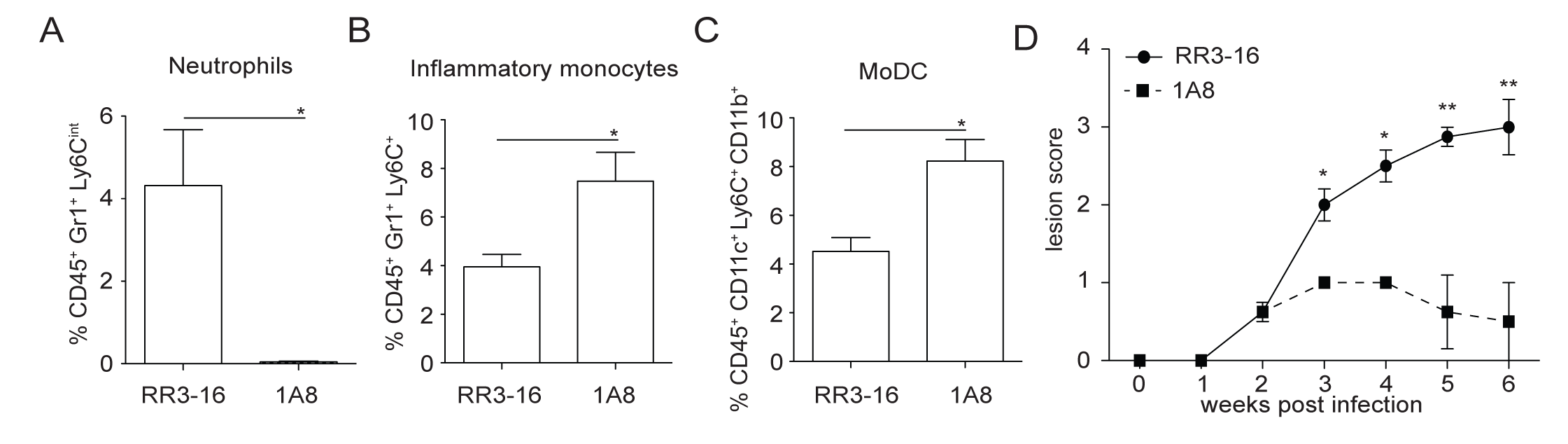

Supplement: S6 Fig — RR3-16-treated and 1A8 PMN-depleted BALB/c mice were inoculated i.d. with 106 metacyclic L. mexicana-DsRed parasites. Ear cells were isolated 3 days following infection and the frequency of (A) CD45+Gr1+Ly6Cint neutrophils (B) CD45+Gr1+Ly6C+ inflammatory monocytes and (C) CD45+CD11c+Ly6C+CD11b+ MoDCs was analyzed by flow cytometry. Quantitation in frequency is given and presented as the mean ± SEM (n = 4/group). (D) Impact of early neutropenia on lesion development following i.d. inoculation of 106 metacyclic L. mexicana in BALB/c mice. BALB/c mice were treated with the 1A8 PMN-depleting mAb resulting in neutropenia during the first three days of infection or with the control RR3-16 mAb. Ear lesion scores were measured on a weekly basis as described in the Materials and Methods. Results are presented as mean lesion score ± SEM * p<0.05 ** p<0.005. (TIF) [file ppat.1004929.s006.tif]
